# Non-falciparum malaria imported mainly from Africa: a review from a Portuguese hospital

**DOI:** 10.1186/s12936-017-1952-3

**Published:** 2017-07-25

**Authors:** Rogério Ruas, André Pinto, João Nuak, António Sarmento, Cândida Abreu

**Affiliations:** 1 Infectious Diseases Department, Centro Hospitalar São João, Alameda Professor Hernani Monteiro, 4200 Porto, Portugal; 2 Instituto de Inovação e Investigação em Saúde (I3s), Grupo de I&D em Nefrologia e Doenças Infeciosas, Department of Medicine, Faculty of Medicine, University of Porto, Porto, Portugal

**Keywords:** Non-falciparum malaria, Epidemiology, Imported

## Abstract

**Background:**

Non-falciparum malaria (NFM) has been reported to be responsible for around 25% of imported malaria cases in Europe but is often neglected due to its less severe clinical course when compared to *Plasmodium falciparum.* Differentiation between species is however crucial for a correct approach. The objective of this study is to report the cases of this often missed aetiology of malaria in a tertiary hospital in Portugal.

**Methods:**

Data were retrospectively analysed from patients admitted from January 2006 to August 2016 with a NFM diagnosis based on microscopy, rapid diagnostic tests (RDT) (BinaxNow^®^) and/or PCR. Epidemiologic and clinical aspects were reviewed.

**Results:**

A total of 19 NFM cases were diagnosed, corresponding to 8.4% of the total 225 cases of malaria. Seventeen (89%) were male with a median age of 41 years. All but one case were imported from sub-Saharan Africa, with 12 (63%) of the cases returned from Angola. Microscopy was positive for all patients and correctly identified the species in 12 (63%) patients. BinaxNOW^®^ was performed in all patients and it was positive in 11 cases, showing a sensitivity of 58%. PCR was performed in nine patients and was positive in eight of them, being responsible for the identification of the species in four cases. *Plasmodium malariae* accounted for 37% (n = 7) of the cases, *Plasmodium ovale* for 32% (n = 6) and *Plasmodium vivax* for 17% (n = 3). In three (16%) patients, morphology was suggestive of *P. vivax* or *P. ovale*, but precise species identification was not possible. Regarding presentation, fever was the most reported symptom, and the most frequent laboratory finding was thrombocytopaenia. Quinine–doxycycline was prescribed in eleven patients (58%), chloroquine in six cases (32%) and artemether–lumefantrine in two (11%). All of the patients showed clinical improvement.

**Conclusions:**

NFM remains an important cause of imported malaria in patients from sub-Saharan Africa, alone or as mixed infection with *P. falciparum*. Access to PCR techniques facilitates diagnosis, as low sensitivity from RDTs and microscopy are to be expected.

## Background

Malaria remains one of the most important infectious diseases responsible for a high morbidity and mortality burden worldwide. Nearly half of the world’s population lives in endemic regions [1], and in developed countries malaria is one of the most common causes of fever in migrants and travellers arriving from the tropics [2], constituting a global public health problem. In Portugal, for historical reasons, a close relationship with Portuguese-speaking African countries (Angola, Guinea-Bissau, Mozambique) is observed and imported malaria cases represent a significant burden.

The disease in humans is caused by several species of the genus *Plasmodium. Plasmodium falciparum* is responsible for the majority of cases and is also associated with a more severe course of illness. As such, it is the most studied species in scientific literature. Non-falciparum malaria (NFM), i.e., *Plasmodium vivax, Plasmodium ovale, Plasmodium malariae,* and *Plasmodium knowlesi*, is less well documented, although it has been reported to be responsible for around 25% of the imported malaria cases in Europe [3–5]. *Plasmodium vivax* is the most geographically widespread species, accounting for 6% of estimated cases globally [1]. Outside of Africa, where infection transmission is generally low and seasonal, *P. vivax* comprises about half of the estimated cases [1], with around the same prevalence as *Plasmodium falciparum*. In sub-Saharan Africa, *P. vivax* infections are uncommon because of the presence in a high proportion of the population of Duffy antigen-negative red cells which cannot be invaded by this parasite [6]. *Plasmodium malariae* and *P. ovale* are relatively common in many parts of sub-Saharan Africa and comprise <10% of isolates [1]. *Plasmodium knowlesi* has recently been identified as the cause of human malaria in Malaysian Borneo, but seems so far to be limited to Southeast Asia [7]. Clinically, *P. vivax* and *P. ovale* have a similar presentation to *P. falciparum*, although usually less severe. These two species have dormant liver stages (hypnozoites), and can induce re-activation of malaria several months after the initial infection. *Plasmodium malariae* (quartan fever) can result in long-lasting infection if not well treated. *Plasmodium ovale* and *P. malariae* are also believed to be responsible for asymptomatic cases [8]. Diagnosis of malaria by microscopic examination of Giemsa-stained thick and thin blood films is still considered the gold standard, as it has good sensitivity and specificity and it allows species differentiation and parasitaemia quantification. However, misclassification of non-falciparum species is common and highly dependent on laboratory experience, especially when dealing with very low parasite densities [9]. Rapid diagnostic tests (RDTs) have shown great sensitivity and specificity for *P. falciparum* infection [10–13], but seem to have a limited role in diagnosing non-falciparum species, probably because of small quantities of antigen circulating due to low parasitaemia. BinaxNOW^®^, a RDT available for use in Portugal, detects the histidine-rich protein 2 (HRP-2), specific to *P. falciparum*, and aldolase, a pan-malarial antigen common to all *Plasmodium* species [14], and as such, it is inappropriate to distinguish between non-falciparum species. Polymerase chain reaction (PCR) seems to be the most accurate method of detecting *Plasmodium* spp. and differentiating species [15, 16], although it is not used in routine clinical care due to elevated costs and need of specific techniques.

Due to current incomplete data on artemisinin combination therapy (ACT) efficacy for NFM in non-endemic settings and the need for effective elimination of hypnozoites, differentiation between species is crucial, as well as understanding the epidemiology of NFM. Therefore, the objective of this study was to describe the clinical and epidemiological features of imported cases of NFM in a tertiary hospital centre in Porto, which is the reference centre for malaria and imported diseases in the north of Portugal.

## Methods

Data were retrospectively analysed from patients admitted at São João Hospital Centre (SJHC) from January 2006 to August 2016 with a NFM diagnosis. Parasitological diagnosis was done either by light microscopic observation of Giemsa-stained thin blood smear, immunochromatographic assay for the qualitative detection of *Plasmodium* antigens (BinaxNOW^®^) or by molecular techniques (in-house one step Real Time Polymerase Chain Reaction (PCR) based on the 18S subunit of the parasite’s ribosomal DNA) which became available in 2013. The different *Plasmodium* species were identified morphologically by the laboratory when possible. Demographic, clinical and epidemiological data of the patients were collected. The delay before diagnosis was defined as the time between the onset of symptoms and the day of diagnosis. SPSS was used on data analysis. The study was approved by the SJCH Ethics Committee.

## Results

A total of 19 NFM cases were diagnosed at SJHC, corresponding to 8.4% of a total 225 cases of malaria diagnosed in the same period. *Plasmodium malariae* accounted for 37% (n = 7) of the cases; *P. ovale* occurred in 32% (n = 6), and *P. vivax* in 16% (n = 3). In three (16%) patients the diagnosis of NFM was made by microscopy because of morphology suggestive of *P. vivax* or *P. ovale*, but precise species identification was not possible. During the same period there were 2 cases of mixed infection with *P. falciparum* (one patient with *P. malariae* infection and another with *P. vivax*/*P. ovale*).

Microscopy was positive for all 21 patients. Species identification was made by microscopic examination of Giemsa-stained thin blood films in 12 (63%) patients. Out of the seven cases where species identification was not possible by microscopy, PCR was diagnostic in four patients: three patients as *P. malariae* and one patient as *P. ovale*. In the three remaining patients, species identification was not possible, but BinaxNOW^®^ was positive for the pan-malarial antigen aldolase for two of them, while the other one had a negative RDT. BinaxNOW^®^ was performed in all 19 patients. It was positive for the pan-malarial antigen in 11 (58%) patients. The test was negative in eight (38%) cases, showing a sensitivity of 58% (95% confidence interval 33.5–79.7%). PCR was performed in nine patients and was positive in eight cases (in five patients for *P. ovale* and in three for *P. malariae*). In one patient, PCR was negative for *P. falciparum*, *P. ovale* and *P. vivax* (PCR for *P. malariae* was not performed at that time), although microscopic examination of thin blood films identified the species as *P. ovale.* Out of the seven *P. malariae* cases, four (57%) were not identified by microscopy, needing PCR for the species identification.

Of the studied sample, 89% (n = 17) were male with a median age of 41 years (range 25–68 years). None of the patients was under chemoprophylaxis for malaria at time of infection, and six (32%) patients had a previous history of malaria infection. Seventeen (89%) patients were Portuguese, and the remaining two were from Brazil and Angola. Most of the cases were imported from Angola (63%, n = 12), followed by Equatorial Guinea with three patients and Mozambique with two. One case was imported from outside of Africa, diagnosed as *P. vivax* infection in a patient returning from South America. The remaining case was imported from the Democratic Republic of Congo. Demographic and diagnostic features are resumed in Table 1.

**Table 1:** Demographic and diagnostic features of patients with non-falciparum malaria (2006–2016)

Pt	Gender, age	Date	Origin	NFM	Microscopy	BinaxNow^®^	PCR	Treatment
1	M, 34	2006	Angola	*P. malariae*	*P. malariae*	−	NA	QD
2	F, 29	2010	South America	*P. vivax*	*P. vivax*	+	NA	QD
3	M, 43	2011	Angola	*P. ovale/P. vivax*	Non-falciparum	−	NA	QD
4	M, 36	2011	Angola	*P. malariae*	*P. malariae*	+	NA	CQ
5	M, 47	2011	Mozambique	*P. vivax*	*P. vivax*	+	NA	CQ
6	M, 52	2011	Angola	*P. ovale/P. vivax*	Non-falciparum	+	NA	QD
7	M, 47	2013	Angola	*P. malariae*	*P. malariae*	−	NA	QD
8	M, 43	2013	Angola	*P. ovale*	*P. ovale*	−	*P. ovale*	CQ
9	M, 33	2013	Equatorial Guinea	*P. malariae*	Non-falciparum	+	Positive for *P.* spp., negative for *P. falciparum, P. ovale* and *P. vivax*	QD
10	M, 46	2013	Angola	*P. ovale*	*P. ovale*	+	Negative for *P. falciparum, P. ovale* and *P. vivax*	QD
11	M, 68	2014	Angola	*P. ovale*	Non-falciparum	+	*P. ovale*	QD
12	M, 40	2014	Mozambique	*P. vivax*	*P. vivax*	−	NA	QD
13	M, 38	2015	Angola	*P. ovale*	*P. ovale*	+	*P. ovale*	AL
14	M, 41	2015	Angola	*P. malariae*	Non-falciparum	+	*P. malariae*	CQ
15	M, 49	2015	Equatorial Guinea	*P. ovale*	*P. ovale*	−	*P. ovale*	CQ
16	M, 30	2015	Angola	*P. malariae*	*P. malariae*	−	NA	QD
17	M, 38	2015	E. Guinea	*P. ovale*	*P. ovale*	−	*P. ovale*	AL
18	M, 52	2016	DRC	*P. ovale/P. vivax*	Non-falciparum	+	NA	QD
19	F, 25	2016	Angola	*P. malariae*	Non-falciparum	+	*P. malariae*	CQ

*NA* not applicable, *CQ* chloroquine, *QD* quinine/doxycycline, *AL* artemether/lumefantrine, *DRC* Democratic Republic of Congo

The median number of days between individuals arriving in Portugal and the time of symptom onset was two, ranging from 0 to 30 days in a patient with *P. malariae* infection. The median of delay before diagnosis was seven (2–14) days, with *P. malariae* associated with a longer delay. Clinically, fever was the most reported symptom, present in all patients, followed by chills, headaches and myalgia with 63, 52 and 53%, respectively. One patient presented with acute respiratory distress syndrome (ARDS), a severe malaria according to WHO criteria [17], and was admitted to the Infectious Diseases Intensive Care Unit (ICU). This patient also presented with a suspected pulmonary infection.

All patients presented with thrombocytopaenia (defined as platelets below 150 cells/cu mm) with a mean platelet count of 94 cells/cu mm. Five (26%) had platelets below 50,000, and 47% (n = 9) had platelets below 80,000. Mean haemoglobin levels at admission was 14.5 g/dL with eight patients (42%) presenting with anaemia (defined as haemoglobin below 13 g/dL). Eleven patients (58%) presented with elevation of liver enzymes with a mean alanine aminotransferase (ALT) of 63 U/L (range 17–357), but only seven (37%) had total bilirubin elevated. All cases at admission had elevated C-reactive protein (CRP) (mean level of 118 mg/L), and three patients (24%) had coagulopathy. The mean duration of hospitalization was 5 days (2–15), with no significant differences between species.

Quinine–doxycycline (QD) was the treatment used in 11 patients (58%), followed by chloroquine (CQ) prescribed in six cases (32%) and artemether–lumefantrine (AL) used in two patients (11%). In four patients (21%), QD or AL was initially prescribed before switching to CQ. The patient admitted to the ICU was treated with intravenous QD. Of the 13 patients with *P. vivax*, *P. ovale*, mixed infection, or undetermined species infection, nine (69%) received terminal prophylaxis with primaquine, after exclusion of deficiency of glucose-6-phosphate dehydrogenase (G6PD). The patients who did not receive eradication treatment were either lost to treatment or returned to endemic areas. There were no fatalities and all of the patients showed clinical cure at the time of hospital discharge.

## Discussion

Most scientific literature has focused on falciparum malaria over the years. Due to a lower prevalence and morbidity, data on NFM, especially imported cases, are limited. However, globalization and the current growing migration movements have forced non-endemic countries to become familiar with these diseases, changing the perspective on its ‘lower prevalence’. The clinical differences between falciparum and non-falciparum malaria, as well as the need for terminal prophylaxis in some cases, make the differential diagnosis vital for a correct medical approach of these imported cases. As such, diagnostic difficulties must be overcome to achieve a correct identification of these species.

Scientific literature has reported NFM to be responsible for around 25% of the imported malaria cases in Europe [3–5]. In this study, NFM was responsible for 8.4% of malaria cases, a lower incidence perhaps explained by Portugal’s close relationship to African countries and therefore higher *P. falciparum* incidence. *Plasmodium vivax* has consistently been reported as the most frequent imported NFM infection in Europe [18, 19], as is expected due to its higher global prevalence. However in this study, *P. malariae* was the most frequently identified NFM species, accounting for 37% of the cases. Considering that 95% of the cases were imported from Africa the higher prevalence of non-vivax species is comprehensible. The *Plasmodium* species identification remained to be known in three cases (14%) as microscopy could not differentiate between *P. vivax* and *P. ovale* and PCR was not available in these patients.

Microscopic diagnosis was positive in all patients, but it was able to correctly identify the species in only 63% of the cases. This is explained by different experienced technicians involved at the time of diagnosis. In three cases *P. vivax*/*ovale* differentiation was not possible and most *P. malariae* cases (57%) were not correctly identified by microscopy probably due to the more difficult microscopic identification of this species. BinaxNOW^®^ showed a sensitivity of 58% for NFM infection, concordant with published data [11, 20]. The results were similar between species (*P. malariae* 57%, *P. vivax* 66%, *P. ovale* 50%). PCR has only been available since 2013 and is usually performed when doubts about the microscopic diagnosis are raised either by the microbiologist or the clinician. It was performed in only nine patients and it was positive in eight patients. The one case with negative PCR was identified as a *P. ovale* infection by microscopy, but PCR was negative for *P. falciparum, P. vivax and P. ovale*. At that time, PCR for *P. malariae* was not performed at SJHC, meaning that this case could have been a *P. malariae* infection misdiagnosed by microcopy as *P. ovale*, as normally we would expect a higher sensitivity with PCR techniques [15, 16]. These results show the importance of complementing microscopy observation with PCR for the diagnosis of NFM, especially in settings of less experienced microscopists. As for RDTs, because of their unsatisfactory sensitivity, they do not seem to contribute much to diagnosis of NFM.

The most frequently reported symptoms were fever, chills, headaches, and myalgia, meaning the clinical presentation is indistinguishable from falciparum malaria, although the less severe course of illness could suggest a NFM infection. The longer delay to diagnosis is also suggestive of NFM infection, as it is probably due to less severe clinical symptoms, especially when considering *P. malariae* infection. Late onset of symptoms may also contribute to a longer delay to diagnosis, since the association between disease and tropical setting fades over time in the minds of the patient and the physician. Haematological complications were frequent, and thrombocytopaenia was the most frequent haematologic laboratory finding, present in all patients, while anaemia was only present in 42% of the cases. In this study, only one patient presented with severity criteria defined by the World Health Organization (WHO) for *P. falciparum* [17], most likely due to bacterial coinfection. However, the need remains to adapt these criteria for NFM.

CQ remains the recommended treatment for NFM [17]. However, due often to an initial doubt on the species and fear of more severe infection by *P. falciparum*, QD was the most prescribed treatment in this study. This probably also reflects that physicians are less experienced in NFM, and thus more comfortable in treating falciparum malaria. AL, the ACT regimen available at SJHC since 2013, will probably be more frequently used in the future due to its pan-malarial efficacy. Countries with CQ-resistant *P. vivax*, such as Indonesia, are already defending an ACT-based treatment for all species. A recent systematic review showed efficacy comparable to CQ, but cost-effectiveness studies are needed [21]. Although ACT regimens solve the problem of mixed or undetermined species infection, they have the same limitation as CQ: they are not active on hypnozoites and thus cannot achieve a radical cure. As such, terminal prophylaxis with primaquine is still needed after initial treatment. This reinforces the need for a correct diagnosis and identification of species to prevent malaria relapse and complications.

## Conclusions

NFM remains a neglected tropical disease due to its lesser severity compared to falciparum malaria. However, globalization has forced non-endemic countries to become more familiar with the correct diagnosis and treatment of this infection. In Portugal, NFM is an important cause of imported malaria, even in patients from sub-Saharan Africa. There are still many obstacles to overcome, as correct identification of *Plasmodium* species is not always achieved. In the future, ‘one treatment for all’ strategies based on ACT regimens may simplify therapeutic options, but due to the ability of some species to produce dormant liver stages and cause reactivation of the disease, correct diagnosis and identification of species remains crucial to reduce morbidity. Access to PCR techniques facilitates diagnosis, as microscopy (with low parasitaemia) and RDTs have lower sensitivity than in *P. falciparum* infection.

## References

[1] WHO. World Malaria report 2015. Geneva: World Heath Organization; 2015. http://www.who.int/malaria/publications/world-malaria-report-2015/report/en.

[2] Kain KC, Keystone JS (1998). Malaria in travelers. Epidemiology, disease, and prevention. Infect Dis Clin North Am.

[3] Gautret P, Schlagenhauf P, Gaudart J, Castelli F, Brouqui P, von Sonnenburg F (2009). Multicenter EuroTravNet/GeoSentinel study of travel-related infectious diseases in Europe. Emerg Infect Dis.

[4] Jelinek T (2007). Imported falciparum malaria in Europe: 2007 data from TropNetEurop. Euro Surveill.

[5] Warne B, Weld LH, Cramer JP, Field VK, Grobusch MP, Caumes E (2014). Travel-related infection in European travelers, EuroTravNet 2011. J Travel Med..

[6] Antinori S, Galimberti L, Milazzo L, Corbellino M (2012). Biology of human malaria Plasmodia including *Plasmodium knowlesi*. Mediterr J Hematol Infect Dis..

[7] Singh B, Kim Sung L, Matusop A, Radhakrishnan A, Shamsul SS, Cox-Singh J (2004). A large focus of naturally acquired *Plasmodium knowlesi* infections in human beings. Lancet.

[8] Doderer-Lang C, Atchade PS, Meckert L, Haar E, Perrotey S, Filisetti D (2014). The ears of the African elephant: unexpected high seroprevalence of *Plasmodium oval*e and *Plasmodium malariae* in healthy populations in Western Africa. Malar J..

[9] Kilian AH, Metzger WG, Mutschelknauss EJ, Kabagambe G, Langi P, Korte R (2000). Reliability of malaria microscopy in epidemiological studies: results of quality control. Trop Med Int Health..

[10] De Monbrison F, Gerome P, Chaulet JF, Wallon M, Picot S, Peyron F (2004). Comparative diagnostic performance of two commercial rapid tests for malaria in a non-endemic area. Eur J Clin Microbiol Infect Dis.

[11] Dimaio MA, Pereira IT, George TI, Banaei N (2012). Performance of BinaxNOW for diagnosis of malaria in a US hospital. J Clin Microbiol.

[12] Durand F, Crassous B, Fricker-Hidalgo H, Carpentier F, Brion JP, Grillot R (2005). Performance of the Now Malaria rapid diagnostic test with returned travellers: a 2-year retrospective study in a French teaching hospital. Clin Microbiol Infect.

[13] Stauffer WM, Cartwright CP, Olson DA, Juni BA, Taylor CM, Bowers SH (2009). Diagnostic performance of rapid diagnostic tests versus blood smears for malaria in US clinical practice. Clin Infect Dis.

[14] Khairnar K, Martin D, Lau R, Ralevski F, Pillai DR (2009). Multiplex real-time quantitative PCR, microscopy and rapid diagnostic immuno-chromatographic tests for the detection of *Plasmodium* spp.: performance, limit of detection analysis and quality assurance. Malar J..

[15] Hanscheid T, Grobusch MP (2002). How useful is PCR in the diagnosis of malaria?. Trends Parasitol..

[16] Snounou G, Viriyakosol S, Zhu XP, Jarra W, Pinheiro L, do Rosario VE (1993). High sensitivity of detection of human malaria parasites by the use of nested polymerase chain reaction. Mol Biochem Parasitol.

[17] WHO. Guidelines for the treatment of malaria. 3rd ed. Geneva: World Health Organization; 2015. 26020088

[18] Bottieau E, Clerinx J, Van Den Enden E, Van Esbroeck M, Colebunders R, Van Gompel A (2006). Imported non-*Plasmodium falciparum* malaria: a five-year prospective study in a European referral center. Am J Trop Med Hyg.

[19] Demaison X, Rapp C, de Laval F, Simon F (2013). Malaria attacks due to *P. vivax* or *P. ovale* in two French military teaching hospitals (2000 to 2009). Med Mal Infect..

[20] Farcas GA, Zhong KJ, Lovegrove FE, Graham CM, Kain KC (2003). Evaluation of the BinaxNOW ICT test versus polymerase chain reaction and microscopy for the detection of malaria in returned travelers. Am J Trop Med Hyg.

[21] Visser BJ, Wieten RW, Kroon D, Nagel IM, Belard S, van Vugt M (2014). Efficacy and safety of artemisinin combination therapy (ACT) for non-falciparum malaria: a systematic review. Malar J..

